# An Efficiency Comparison of Document Preparation Systems Used in Academic Research and Development

**DOI:** 10.1371/journal.pone.0115069

**Published:** 2014-12-19

**Authors:** Markus Knauff, Jelica Nejasmic

**Affiliations:** Department of Psychology, Experimental Psychology and Cognitive Science, University of Giessen, Giessen, Germany; University of North Carolina at Charlotte, UNITED STATES

## Abstract

The choice of an efficient document preparation system is an important decision for any academic researcher. To assist the research community, we report a software usability study in which 40 researchers across different disciplines prepared scholarly texts with either Microsoft Word or LaTeX. The probe texts included simple continuous text, text with tables and subheadings, and complex text with several mathematical equations. We show that LaTeX users were slower than Word users, wrote less text in the same amount of time, and produced more typesetting, orthographical, grammatical, and formatting errors. On most measures, expert LaTeX users performed even worse than novice Word users. LaTeX users, however, more often report enjoying using their respective software. We conclude that even experienced LaTeX users may suffer a loss in productivity when LaTeX is used, relative to other document preparation systems. Individuals, institutions, and journals should carefully consider the ramifications of this finding when choosing document preparation strategies, or requiring them of authors.

## Introduction

The key communication of academic research and development is through diverse forms of publications. Most scholars spend many hours writing journal articles, books, or other forms of scholarly text. Virtually all researchers use one of two document preparation systems: Microsoft Word or LaTeX. Publishers often accept just one of the two text file formats [1]. Microsoft Word is based on a principle called “What you see is what you get” (WYSIWYG), which means that the user immediately sees the document on the screen as it will appear on the printed page. LaTeX, in contrast, embodies the principle of “What you get is what you mean” (WYGIWYM), which implies that the document is not directly displayed on the screen and changes, such as format settings, are not immediately visible. Microsoft Word requires little start-up time and provides easy and instantaneous control of textual input and output. Microsoft Word is the predominant document preparation system across many disciplines, including medicine, law, business, and the life sciences, and is also the dominant document preparation system for professional communications. LaTeX, in contrast, is a programming language that requires the use of an external editing interface to produce documents. LaTeX is frequently used in mathematics, physics, computer science, and engineering because it provides the user unlimited flexibility and is particularly useful if the user needs to set complex mathematic equations in a professional layout. LaTeX is freely available as open-source software. In contrast, Microsoft Word is a commercial product licensed by the Microsoft Corporation.

In the “publish or perish” age of academic research, many senior researchers advise their students and junior researchers about how to create professional document layouts, which software system to use, and which system is more efficient or user-friendly. Many of these senior researchers will attempt to convince their students and junior researchers that one system is “better”, “more elegant” “simpler”, or “more flexible” than the other system. There are very few researchers, however, who can confirm empirically how one system is superior to the other and on what basis they have drawn this conclusion. To date, no empirical studies exist to identify which system is more efficient. The preference toward a particular document preparation system can be particularly obstructive to the progress of research if the research question requires interdisciplinary teams. For example, a brain computer interface project may require collaborations between medical scientists, psychologists, computer scientists, biologists, physicists, and engineers. Any researcher who has ever collaborated on such large interdisciplinary projects has experienced the difficulty with reaching a consensus about which document preparation system to use. Discussions about document preparation systems are often unproductive and driven by preconceived opinions, individual biases, and disciplinary traditions. A fair comparison of the efficiency and usability of the different document preparation systems based on empirical evidence rather than individual habits and biases may facilitate such discussions.

## Participants, Materials, and Methods

To assist the academic research community in the choice of an efficient document preparation system, we empirically compared the usability of LaTeX and Word under highly realistic working conditions. The volunteers for this study included 40 researchers and advanced graduate students from six German universities who wrote scholarly texts in either Microsoft Word or LaTeX (mean age 25.4 years; 14 female; Physics: 12; Psychology: 5; Computer Science: 4; Mathematics: 4; Electrical engineering: 3; MBA: 3; Sport Science: 4; others: 5). They were recruited from newsgroups, mailing lists, blogs, and other sources. Most participants were tested in their personal office setting, and all participants used their own computer, which ran either the Windows or Linux operating system. They were informed that the purpose of the study was to evaluate the quality of their document preparation system they use in their daily work.

All participants were properly instructed and have indicated that they consent to participate by signing the informed consent paperwork. The study has been conducted according to the principles expressed in the Declaration of Helsinki; the risks of the study were no higher than those experienced by people using their respective software (Word or LaTeX) on a day-to-day basis, participants could withdraw from the task at any time, and no identifiable data will be released about participants. For such studies the ethical guidelines of the Deutsche Gesellschaft für Psychologie (German Psychological Society, DGPs) and the Bund Deutscher Psychologen (German Psychological Association, BDP) revised on June 28, 2004 specify that approval from an Ethics Committee can be waived “if it can reasonably be assumed that participation in the research produces no damage or no discomfort that go beyond everyday experience, and if the research (a) refers to common education methods, curricula or teaching methods in education; … or (c) refers to factors that affect work and organizational efficiency in organizations whose investigation can have no occupational disadvantages for individuals and for which confidentiality is guaranteed (p. 2, paragraph 6)”. The present research belongs to this class of studies; thus, no further approval from an ethics committee was required.

The participants were divided into 4 groups with 10 participants in each group: Word novices, Word experts, LaTeX novices, and LaTeX experts. Participants were classified as “novices” if they had less than 500 hours of experience with the respective program and “experts” if they had more than 1000 hours of experience with the respective program. In the resulting groups, participants who were classified as “novices” had on average 234 hours (*SD* = 153) experience with the respective program, whereas “experts” had on average 1909 hours experience with the respective program (*SD* = 211).

**Fig 1:**
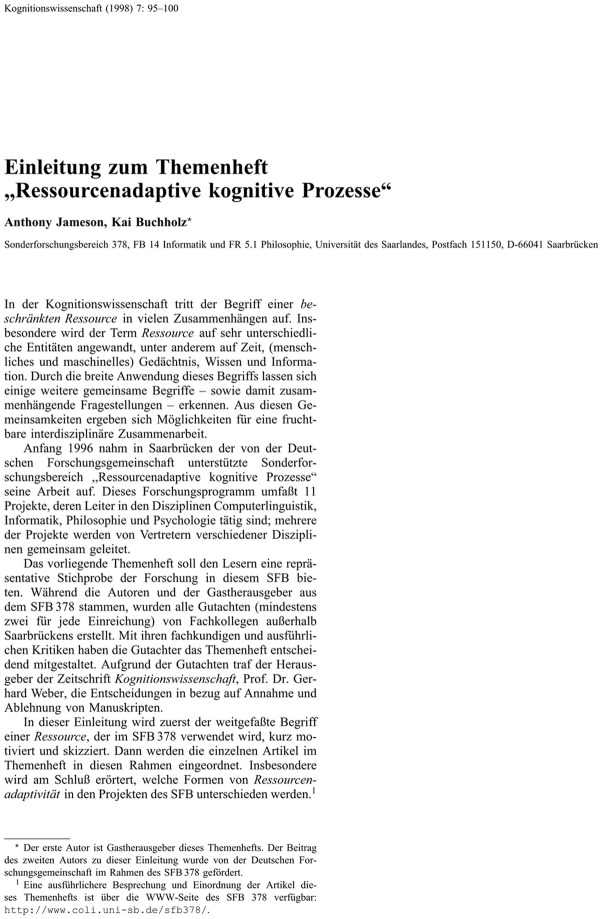
The *continuous text* used in the present study. From: Jameson, A. & Buchholz, K. (1998). Einleitung zum Themenheft “Ressourcenadaptive kognitive Prozesse”, *Kognitionswissenschaft*, 7, 95.

**Fig 2:**
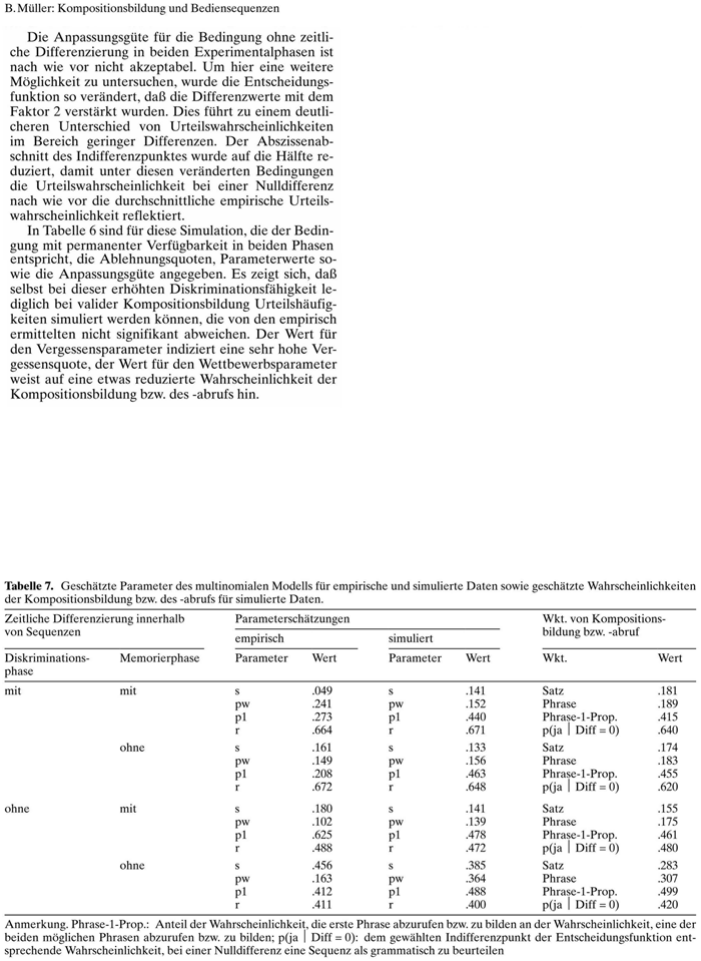
The *table text* used in the present study. From: Müller, B. (1998). Kompositionsbildung bei Symbolfolgen und Bediensequenzen: Empirische Befunde und die Theorie des “Competitive Chunking”, Kognitioswissenschaft, 7, 85.

**Fig 3:**
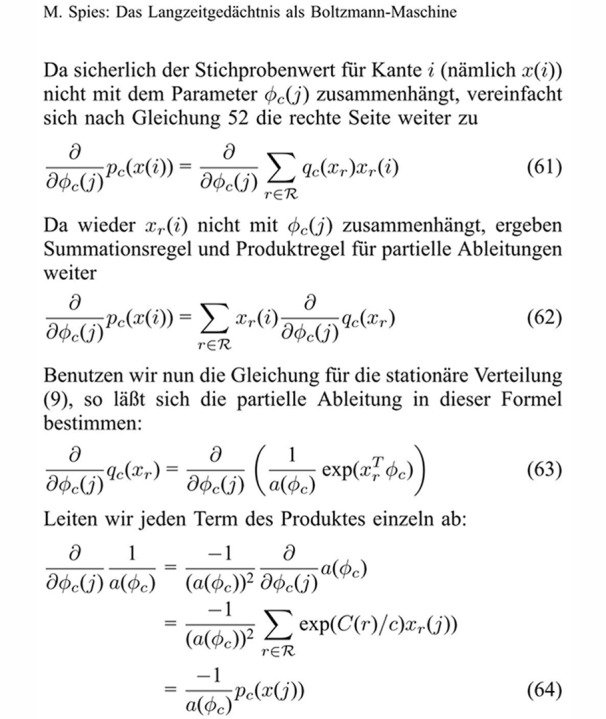
The *equation text* used in the present study. From: Spies, M. (1999). Das Langzeitgedächtnis als Boltzmann-Maschine, *Kognitionswissenschaft*, 8, 71.

The probe texts included three different text structures: (1) simple continuous text; (2) text with tables; and (3) mathematical text with several equations. The texts were selected based on a pilot study so that an expert could reproduce around 90% of the text in thirty minutes. All texts came from the Journal “Kognitionswissenschaft” which was the official Journal of the German Cognitive Science Society until the year 2002. The selected texts are presented in Fig. 1. The continuous text consisted of a headline and headings with different font sizes, four paragraphs, and two footnotes (Fig. 1). The table text consisted of a headline, two paragraphs, and a table that was divided into several segments and surrounded by text (Fig. 2). The equation text consisted of a headline, four paragraphs, and six equations (Fig. 3). Participants were allowed to use all tools, editors, plug-ins, and add-ons that they were accustomed to using with their respective software. For example, many LaTeX users produce documents with external text editors such as TeXnicCenter, LaTeX Editor, Kile, or WinEdit because LaTeX does not offer an internal text editor. All participants already had some experience with formatting tables and equations and were tested in the presence of the experimenter. The three text types were presented in a random order to each participant. The participants were instructed to reproduce the source text within thirty minutes. Each participant was given five minutes to familiarize themselves with the text. The performance of each participant was measured for each text sample by three variables: (1) the number of orthographic and grammatical mistakes; (2) the number of formatting errors and typos; and (3) the amount of written text (in symbols and words) produced within 30 minutes. Table 1 provides an overview of all possible errors in the three probe texts. To measure the user’s opinions and satisfaction with their software system, each participant also completed an international standard questionnaire (ISO 9241–10) about usability engineering. To motivate the participants, the best three performers from each group received a monetary prize of 150, 100, or 50 euros, respectively. In the following section, we report the performance of the four groups of participants for the three types of probe text. Then, we report the results of the ISO 9241–10 questionnaire, which examines how well each document preparation system fulfilled the general ergonomic principles that apply to the design of dialogues between humans and information systems. In the final part of the article, we present some psychological explanations for the reported results and discuss some implications for academic research and development.

**Table 1:** Overview of possible mistakes in the three probe texts.

		Continuous text	Table text	Equation text
Orthographic and grammatical mistakes	in words	X	X	X
	in formulas	—	—	X
Formatting errors and typos	header	X	X	X
	headline	X	—	—
	paragraph	X	X	X
	spacing	X	X	X
	font	X	X	X
	footnote	X	—	—
	columns	X	X	X
	lines	—	X	—
	justified text	X	X	X
Amount of written text	missing words	X	X	X
	missing signs	X	X	X

Note: X = possible;— = Not possible

## Results

**Fig 4:**
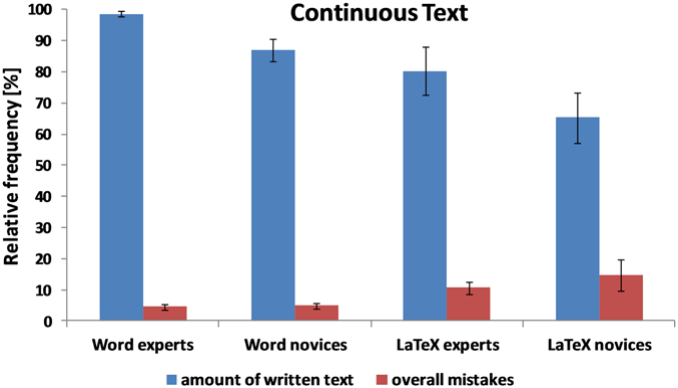
Mean amount of text written within 30 minutes and the overall number of mistakes for the *continuous text* for the four groups of participants (Word experts, Word novices, LaTeX experts, and LaTeX novices). Error bars represent the standard error.

The performance of the four experimental groups (Word novices, Word experts, LaTeX novices, and LaTeX experts) on the three probe texts (continuous text, table text, and equation text) is summarized in Table 2 and Figs. 2, 3, and 4. The results of the usability questionnaire are presented in Table 3.

**Table 2:** Mean absolute frequencies of orthographic and grammatical mistakes, formatting errors and typos, and the amount of written text (i.e., number of words) across all four groups for the continuous text (a), the table text (b), and the equation text (c).

a. Continuous text												
	Word						LaTeX					
	Novices		Experts		Overall		Novices		Experts		Overall	
	*M*	*SD*	*M*	*SD*	*M*	*SD*	*M*	*SD*	*M*	*SD*	*M*	*SD*
Orthographic and grammatical mistakes	5.9	3.5	7.9	6.7	6.9	5.3	7.0	6.6	11.3	9.5	9.2	8.2
Formatting errors and typos	10.0	3.9	9.3	4.1	9.7	3.9	17.3	4.1	16.1	4.0	17.1	4.0
Amount of written text	331	49.1	379	11, 7	355	42.4	250	104	308	99.4	279	103.3

b. Table text												
	Word						LaTeX					
	Novices		Experts		Overall		Novices		Experts		Overall	
	*M*	*SD*	*M*	*SD*	*M*	*SD*	*M*	*SD*	*M*	*SD*	*M*	*SD*
Orthographic and grammatical mistakes	9.9	7.8	7.1	4.4	8.5	6.4	9.7	10.5	7.8	4.5	8.8	7.9
Formatting errors and typos	12.0	3.7	11.3	4.1	11.4	3.9	19.5	3.6	18.7	3.0	19.1	3.3
Amount of written text	353	82.9	395	78.7	374	81.6	191	118	260	137.8	226	130

c. Equation text												
	Word						LaTeX					
	Novices		Experts		Overall		Novices		Experts		Overall	
	*M*	*SD*	*M*	*SD*	*M*	*SD*	*M*	*SD*	*M*	*SD*	*M*	*SD*
Orthographic and grammatical mistakes	5.2	4.1	3.9	3.5	4.6	3.8	11.4	8.2	9.3	7.9	10.4	7.9
Formatting errors and typos	24.4	6.4	19.3	11.8	21.9	9.6	14.9	5.5	12.5	4.9	13.7	5.2
Amount of written text	231	57.4	270	67.3	250	64.1	314	16.7	312	24.6	313	20.5

Note—Orthographic and grammatical mistakes were counted as one mistake per word, even if a participant made more than one mistakes in a word. Each formatting error and each typo was counted as one mistake. For instance, if a text contains three different font sizes each wrong formatted text section was counted as one mistake.

**Table 3:** Results from the usability questionnaire ISO 9241–10.

	Software			
	Word		LaTeX	
Usability questionnaire	*M*	*SD*	*M*	*SD*
Tiredness	3.4	1.9	2.2	1.4
Frustration	3.3	2.0	2.1	1.5
Enjoyment	3.6	1.7	5.2	1.4
Suitability for the task	0.6	1.1	1.4	0.8
Self-descriptiveness	-0.2	0.9	-0.3	1.2
Controllability	1.6	1.0	1.7	0.9
Conformity with user expectations	1.3	0.7	1.3	0.9
Error tolerance	0.3	1.1	-0.6	1.2
Suitability for individualization	0.2	1.1	0.7	1.1
Suitability for learning	0.4	1.1	-0.3	0.8

### Continuous text

As shown in Table 2a and Fig. 4, Word users (both novices and experts) made fewer formatting mistakes (*t* (37.97) = -5.94, *p* <. 001) and wrote significantly more text within 30 minutes (*t* (38) = 3.10, *p* <. 01) compared with LaTeX novice and expert users. The number of orthographic and grammatical errors did not differ significantly between Word and LaTeX users (*t* (38) = -1.02, *p* = .31). However, Word experts made significantly fewer formatting mistakes than LaTeX experts (*t* (18) = -4.15, *p* <. 01) and Word novices made significantly fewer formatting mistakes than LaTeX novices (*t* (17.92) = -4.05, *p* <. 01). Interestingly, Word novices also made significantly fewer formatting mistakes than LaTeX experts (*t* (17.98) = -3.84, *p* <. 01). Word experts wrote significantly more text than LaTeX experts (*t* (18) = 2.24, *p* <. 05) and Word novices wrote significantly more text than LaTeX novices (*t* (18) = 2.31, *p* <. 05).

### Table text

**Fig 5:**
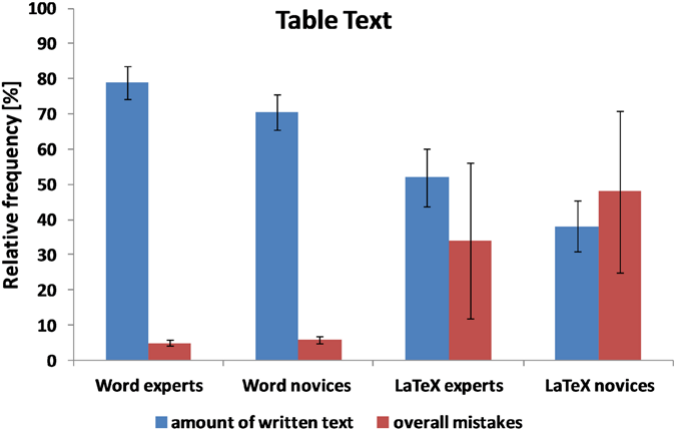
Mean amount of text written within 30 minutes and the overall number of mistakes for the, table text for the four groups of participants (Word experts, Word novices, LaTeX experts, and LaTeX novices). Error bars represent the standard error.

As shown in Table 2b and Fig. 5, Word users (both novices and experts) made significantly fewer formatting mistakes (*t* (36.78) = -6.72, *p* <. 001) and wrote more text within 30 minutes (*t* (31.73) = 4.31, *p* <. 001) compared with LaTeX novice and expert users. Word experts made significantly fewer formatting mistakes than LaTeX experts (*t* (16) = -4.40, *p* <. 001) and Word novices made significantly fewer mistakes than LaTeX novices (*t* (18) = -4.98, *p* <. 001). Word experts wrote significantly more text than LaTeX experts (*t* (14.19) = 2.68, *p* <. 05) and Word novices wrote significantly more text than LaTeX novices (*t* (15.99) = 3.52, *p* <. 01). Interestingly, Word novices made significantly fewer formatting mistakes (*t* (17.33) = -4.78, *p* <. 001) and produced more text than LaTeX experts (*t* (15.99) = 3.52, *p* <. 01).

### Equation text

**Fig 6:**
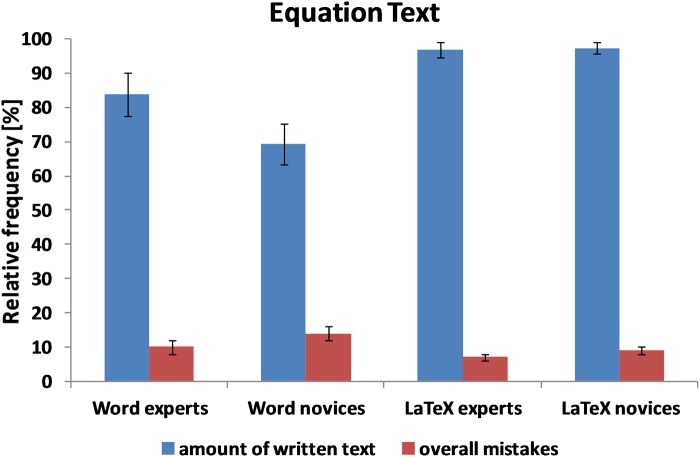
Mean amount of text written within 30 minutes and the overall number of mistakes for the equation text for the four groups of participants (Word experts, Word novices, LaTeX experts, and LaTeX novices). Error bars represent the standard error.

As shown in Table 2c and Fig. 6, LaTeX users (both novices and experts) made significantly fewer formatting mistakes (*t* (38) = 3.35, *p* <. 01) and wrote more text within 30 minutes (*t* (38) = -2.96, *p* <. 001) compared with Word novices and experts. However, LaTeX users made significantly more orthographic and grammatical errors than Word users (*t* (38) = -2.96, *p* <. 01). LaTeX novices made significantly fewer formatting mistakes (*t* (17.61) = 3.57, *p* <. 01) and also wrote more text (*t* (18) = -4.30, *p* <. 001) than Word novices. Overall, however, the performance of LaTeX experts and Word experts did not differ significantly.

### Usability questionnaire

The international standard questionnaire ISO 9241–10 measures user’s opinions and satisfaction with their software system. The questionnaire addresses general ergonomic principles that apply to the design of dialogues between humans and information systems, including suitability for the task, suitability for learning, suitability for individualization, conformity with user expectations, self-descriptiveness, controllability, and error tolerance. Furthermore, we asked whether users perceived their work with the respective software as tiresome, frustrating, or delightful. Participants rated their software on a seven-point scale from very bad (-3) to very good (3). As shown in Table 3, Word users rated their respective software as less efficient than LaTeX users (*t* (35.6) = -2.80, *p* <. 01), but LaTeX users rated the learnability of their respective software as poorer than Word users (*t* (33.6) = 2.13, *p* <. 05). However, LaTeX users assessed their work as less tiresome (*t* (35.38) = 2.16, *p* <. 05) and less frustrating than Word users (*t* (38) = 2.27, *p* <. 05). LaTeX users significantly more often reported to enjoy their work with their respective software than Word users (*t* (36.27) = -3.23, *p* <. 01).

## Discussion

Many academic authors believe that they have the right to design documents themselves and that each researcher should have the freedom to choose the software that he or she prefers. In fact, our study shows that each document preparation system has unique advantages and disadvantages, and there might be no “best” tool for all aspects of a highly complex task such as producing diverse scientific publications. For example, LaTeX users in our study attained better performance in the typesetting of mathematical equations, and it is not surprising that LaTeX users are typically in disciplines where mathematical formulas are frequent (e.g., mathematics, engineering, or computer science). Indeed these disciplinary preferences fit with the original motivation for the development of TeX (the basis of LaTeX) in the 1970s, which was to provide a powerful means to typeset complex mathematical formulas [2]. Some computer scientists may therefore think that mastering LaTeX is a “must” for any “true” expert in their discipline and that someone who already invested significant time and effort in learning LaTeX may not want to re-learn another tool. One may also argue that given a well-designed LaTeX document class file, document development speed and text and formatting accuracy are significantly improved. Another characteristic of our study is that it is practically impossible to evaluate LaTeX without also evaluating the used editors. In fact, our research measured the efficiency of Word against LaTeX in combination with some editor interfaces. However, recent research shows that it is possible to improve the interfaces to LaTeX by making them do more what the authors expect instead of what the programmers imagined [3].

However, our study suggests that LaTeX should be used as a document preparation system only in cases in which a document is heavily loaded with mathematical equations. For all other types of documents, our results suggest that LaTeX reduces the user’s productivity and results in more orthographical, grammatical, and formatting errors, more typos, and less written text than Microsoft Word over the same duration of time. LaTeX users may argue that the overall quality of the text that is created with LaTeX is better than the text that is created with Microsoft Word. Although this argument may be true, the differences between text produced in more recent editions of Microsoft Word and text produced in LaTeX may be less obvious than it was in the past. Moreover, we believe that the appearance of text matters less than the scientific content and impact to the field. In particular, LaTeX is also used frequently for text that does not contain a significant amount of mathematical symbols and formula. We believe that the use of LaTeX under these circumstances is highly problematic and that researchers should reflect on the criteria that drive their preferences to use LaTeX over Microsoft Word for text that does not require significant mathematical representations.

One decision criterion that factors into the choice to use a particular software system is the usability of the available systems for the given task. The usability of a software system is a measure of how easy it is to use the program to carry out a prescribed task. In human-computer interaction and cognitive ergonomics, the most central aspects of usability include the “efficiency” of the system (which refers to how quickly users can perform tasks once they have learned the design), “errors” (which refers to how many errors users make, the severity of these errors, and how easily users can recover from these errors), and “user satisfaction” (the overall pleasantness and feasibility of the design) [4,5]. Based on these criteria, our results show that no reasons exist to use LaTeX for documents that do not contain complex mathematical formula.

A second decision criterion that factors into the choice to use a particular software system is reflection about what drives certain preferences. A striking result of our study is that LaTeX users are highly satisfied with their system despite reduced usability and productivity. From a psychological perspective, this finding may be related to motivational factors, i.e., the driving forces that compel or reinforce individuals to act in a certain way to achieve a desired goal. A vital motivational factor is the tendency to reduce cognitive dissonance. According to the theory of cognitive dissonance, each individual has a motivational drive to seek consonance between their beliefs and their actual actions. If a belief set does not concur with the individual’s actual behavior, then it is usually easier to change the belief rather than the behavior [6]. The results from many psychological studies in which people have been asked to choose between one of two items (e.g., products, objects, gifts, etc.) and then asked to rate the desirability, value, attractiveness, or usefulness of their choice, report that participants often reduce unpleasant feelings of cognitive dissonance by rationalizing the chosen alternative as more desirable than the unchosen alternative [6, 7]. This bias is usually unconscious and becomes stronger as the effort to reject the chosen alternative increases, which is similar in nature to the case of learning and using LaTeX.

A third decision criterion that should factor into a researcher’s choice of a document preparation system is the cost of research and development to the public or industry. Researchers have a responsibility to act economically and efficiently to create new technologies and theories that benefit society, especially in cases in which research is publicly funded. In 2010, the 27 countries of the European Union invested approximately 247 billion euros into research and development, which represents approximately 1.9 percent of the EU’s gross domestic product. In the same year, Germany invested 2.9% (75 billion euros) and the US invested 2.8% (370 billion dollars) of its gross domestic expenditures into research and development. A significant portion of these budgets is allocated to the salaries of researchers. According to the American Association for the Advancement of Science (AAAS), there are approximately 5.8 million science and engineering researchers worldwide [8]. No reliable data is available about how many of these researchers use LaTeX (or MS Word). However, a google search for LaTeX (together with TeX to avoid ambiguities) results in approximately 18 million hits. Brischoux and Legagneux found that approximately 26% of submissions to 54 randomly selected scholarly journals from 15 different scientific disciplines were written in LaTeX, with a significant difference between LaTeX-using and non-LaTeX-using disciplines [1]. We can only roughly estimate the average number of hours per day that a researcher spends on writing scholarly texts, such as internal technical reports, journal articles, and book publications. For researchers in the field of cognitive and brain science, researchers may spend approximately 10 to 30 percent of their time engaged in writing.

Given these numbers it remains an open question to determine the amount of taxpayer money that is spent worldwide for researchers to use LaTeX over a more efficient document preparation system, which would free up their time to advance their respective field. Some publishers may save a significant amount of money by requesting or allowing LaTeX submissions because a well-formed LaTeX document complying with a well-designed class file (template) is much easier to bring into their publication workflow. However, this is at the expense of the researchers’ labor time and effort. We therefore suggest that leading scientific journals should consider accepting submissions in LaTeX only if this is justified by the level of mathematics presented in the paper. In all other cases, we think that scholarly journals should request authors to submit their documents in Word or PDF format. We believe that this would be a good policy for two reasons. First, we think that the appearance of the text is secondary to the scientific merit of an article and its impact to the field. And, second, preventing researchers from producing documents in LaTeX would save time and money to maximize the benefit of research and development for both the research team and the public.

## Supporting Information

S1 Materials Data File of the study. Explanations are provided in S2 Materials.
(XLSX)

S2 Materials Explanations of data file including variable definitions. (TXT)

S3 Materials Additional information. (PDF)
