# Correction: An Efficiency Comparison of Document Preparation Systems Used in Academic Research and Development

**DOI:** 10.1371/journal.pone.0125830

**Published:** 2015-04-23

**Authors:** The PLOS ONE Staff

## Notice of Republication

This article was republished on March 30, 2015, to correct the sizing and placement of the figures; none of the article content was changed. The publisher apologizes for the original layout errors. Please download this article again to view the corrected version. The originally published, uncorrected article and the republished, corrected article are provided here for reference.

## Supporting Information

S1 File Originally published, uncorrected article. (PDF)

S2 File Republished, corrected article. (PDF)
