# Supplementary material for: An Efficiency Comparison of Document Preparation Systems Used in Academic Research and Development
Source: PLoS One. 2014 Dec 19;9(12):e115069. doi: 10.1371/journal.pone.0115069 (PMC4272305; doi:10.1371/journal.pone.0115069)
Supplement: S3 Materials — (PDF) [file pone.0115069.s003.pdf]

Additional Information

An Efficiency Comparison of Document Preparation Systems

used in Academic Research and Development

Markus Knauff & Jelica Nejasmic (2014)

In the introduction of the paper, we state that LaTeX embodies the principle of “What you get is what you mean” (WYGIWYM), which implies that the document is not directly displayed on the screen and changes, such as format settings, are not immediately visible. Although this is true, LateX users often use synchronous-formatting interfaces that display the printed page in standard PDF which, in principle, is more WYSIWYG than Word.

The study was conducted when most of the participants in the Word groups used Word Version 12. Only 3 participants used older versions of Microsoft Word. Newer Versions of Word include a new Equation editor that makes the software even more powerful and easier to use.
